# Neural Monitoring With CMOS Image Sensors

**DOI:** 10.29252/NIRP.BCN.9.3.227

**Published:** 2018

**Authors:** Azar Yadegari, Mohammad Azim Karami, Mohammad Reza Daliri

**Affiliations:** 1. Department of Electrical Engineering, School of Electrical Engineering, Iran University of Science and Technology, Tehran, Iran.

**Keywords:** Image sensor, Implantable devices, Neural monitoring

## Abstract

Implantable image sensors have several biomedical applications due to their miniature size, light weight, and low power consumption achieved through sub-micron standard CMOS (Complementary Metal Oxide Semiconductor) technologies. The main applications are in specific cell labeling, neural activity detection, and biomedical imaging. In this paper the recent research studies on implantable CMOS image sensors for neural activity monitoring of brain are being quantified and reviewed. Based on the results, the suitable implantable image sensors for brain neural monitoring should have high signal to noise ratio of above 60 dB, high dynamic range of near 88 dB and low power consumption than the safety threshold of 4W/cm^2^. Moreover, it is found out that the next generation of implantable imaging device trend should reduce the pixel size and power consumption of CMOS image sensors to increase spatial resolution of sample images.

## Highlights

- The main parameters of image sensors characteristics can be used as suitable candidate for neural monitoring applications.
- The dynamic range and signal to noise ratio evaluation are neccessary for the image sensor design process to be used in neural monitoring applications.

## Plain Language Summary

Neural monitoring systems are used as a new tool for the characterization of the neural behaviors. We reviewd in this article the main parameters which describe the image sensor operation to be useful in neural monitoring systems. Also, the necessary parameters such as dynamic range and signal to noise ratio is quantified in image sensors to be used in neural monitoring applications.

## 1. Introduction

Several applications are introduced for the image sensors in the biomedical engineering such as brain neuronal activity monitoring (Ahmadi & Jullien, 2009), blood glucose self-monitoring (Al-Ashmouny et al., 2009), intrinsic signal detection (Barretto et al., 2011), brain functions fluorescence imaging, the dynamics of cancer cell death monitoring (Bermak, Bouzerdoum, & Eshraghian, 2002), advanced therapies (Brancaleon & Moseley, 2002), capsule endoscopes, and retina prosthesis (Braun & Fromherz, 2004). Image sensors have been developed for implantation in various parts of the human or animal bodies such as eyes (Choi et al., 2013) or brain (Deguchi, Maruyama, Yamasaki, Hamamoto, & Izumi, 1992; Dupret, Tchamgaspanian, Verdant, Alacoque, & Peizerat, 2011). Basically, there are two types of image sensors implanted in the head of human or animal; artificial retinal prosthetic device which has been developed for electrical stimulation of the retina and improve the quality of life for blind patients who have diseases like retinitis pigmentosa and age-related macular degeneration (El Gamal & Eltoukhy, 2005), image sensors proposed for brain imaging. In this paper, the recent research studies on implantable CMOS image sensor for neural activity monitoring of brain are being quantified and reviewed.

Pacemakers are used to cure disturbances such as heart failure, fast heart beating, ventricular fibrillation and stroke. The first implantable pacemaker was developed in 1958 (El-Ali, Sorger, & Jensen, 2006). In addition, image sensors are recently being invoked for various biomedical applications such as brain imaging, control of cells and tissues growth, monitoring of neural activity, improving eyesight, as well as detecting and manipulating individual cells (Fenno, Yizhar, & Deisseroth, 2011; Ferguson & Redish, 2011; Ghovanloo & Atluri, 2007; Greatbatch & Holmes, 1991). In section 2, neural monitoring system with CMOS image sensors for studying neural activity in the brain of mouse is described. Moreover, in section 3, different illumination setups and signal collection methods in implantable neural monitoring system are given. Section 4 briefly describes CMOS image sensors and the main specifications for biomedical applications. Finally, section 5 presents a summarized comparison among some of image sensors in vivo implantation.

## 2. Neural Monitoring System Description With Image Sensors

**Figure 1:**
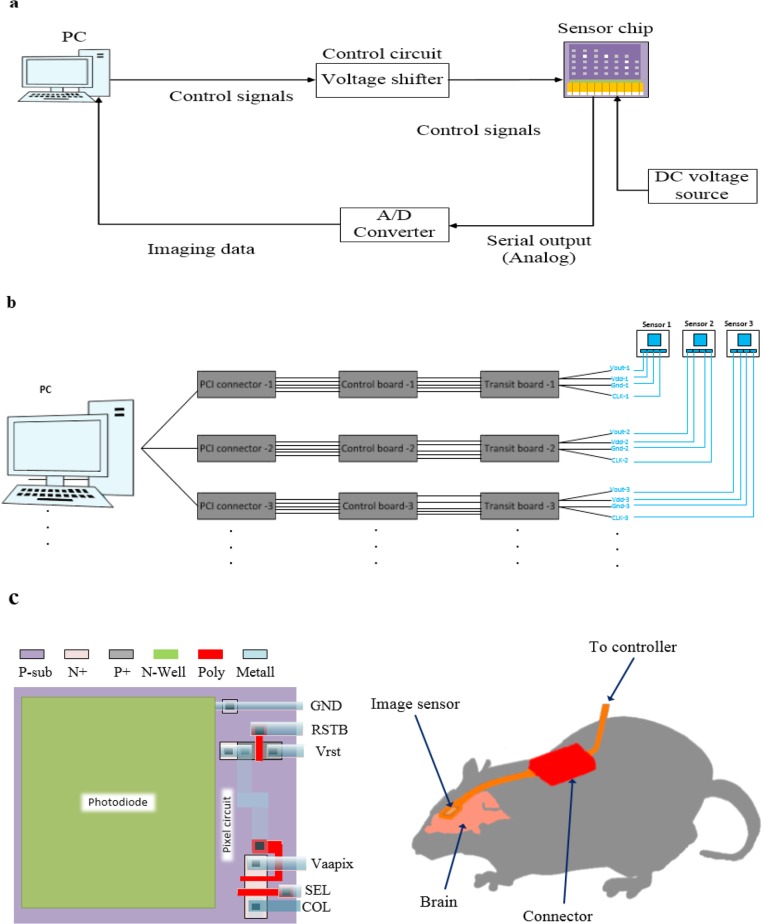
(a) Block diagram of the hardware interface of imaging system; (b) Multiple sensors are linked together to reduce the system size, sharing lines, boards, and connectors; (c) Pixel layout of image sensor and schematic image of an imaging system which is implanted in the mouse’s brain

An imaging system for neural monitoring contains a processing system or a Personal Computer (PC), control board, and Analog to Digital (A/D) converter module. The PC sends control signals to the control board that send the mentioned signals to the image sensor. Since the operating voltage of the image sensor is limited (lower than operating voltage of PC), a special board is required to convert higher voltage signals from the PC to the operating voltage of CMOS image sensor (voltage shifter). The serial output data stream from the image sensor is sent to the A/D converter. Furthermore, digital signal output of A/D (imaging data) is extracted through the Peripheral Component Interconnect (PCI) connector to the PC, as shown in Figure 1(a). It should be noted that the imaging digital data is processed by a software (Hachisuka et al., 2005).

As the number of sensors increases, the overall imaging system becomes noticeably large (Dupret et al., 2011). To reduce the size of imaging system, image sensors are linked together, sharing lines, boards and connectors, as shown in Figure 1(b). The image sensor and Light-emitting Diodes (LEDs) which will be discussed later in this paper are mounted on a flexible polyimide substrate with wire-bonding (Dupret et al., 2011). The excitation light of the LED excites fluorescence from neural cells (Dupret et al., 2011). Basically a power supply source is settled inside the implanted device to supply the LEDs which can consume up to 100 mW/ of power (Haruta et al., 2014). The fluorescence light passes through a red absorption filter which is mounted on top of the pixel array; the absorption filter blocks excitation light. An example for the layout of pixel which can be implanted inside the brain and schematic representation of an implantable imaging device in freely moving mouse is shown in Figure 1(c). An implanted imaging system affects biological tissues of body due to mechanical stress and distortion. Thus, a waterproof and biocompatible packaging is necessary to coat the imaging system (Haruta et al., 2014; Hayami et al., 2016; Ho et al., 2014; Kobayashi et al., 2014; Kobayashi et al., 2011; Koppa, Park, Joo, & Jung, 2011; Lee, Kim, & Youn, 2014; Lin, Lai, & King, 2004).

## 3. Different Illumination Setups and Signal Collection Configurations

Light has a promising potential as a safe energy source for imaging and therapeutic purposes of brain tissues, for Photodynamic Therapy (PDT), light-induced gene transfer, optogenetic neural interface, and optogenetic protein therapy (Loeb, Richmond, & Baker, 2016; Manaresi et al., 2003; Nakamura, 2017; Ng et al., 2008). Several methods have been used to illuminate the brain tissues with optical stimulation systems; transdermal approach for light delivery and detection, fiber-optic device approach, and semiconductor device approach.

**Figure 2:**
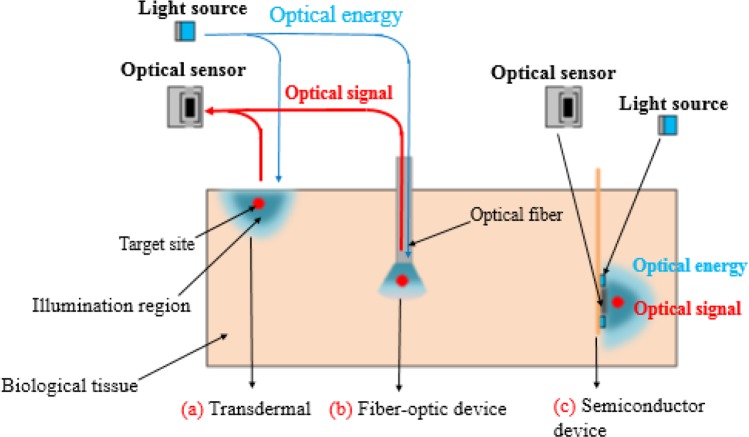
Different approaches to deliver light into and detecting optical signals from outside of the body (a) Transdermal approach for light delivery and detection (head-mountable device); (b) Fiber-optic device approach; (c) Semiconductor device approach (the brain-implantable device)

As shown in Figure 2(a), recently for transdermal light delivery and detection, an external optical monitoring system has been introduced to assess spasticity in a clinical study (Ng et al., 2006). This method demonstrates the efficiency of optogenetic and non-invasive monitoring to evaluate the bioactivity. This optical system is used for imaging targets near the surface of the skin because both of optical source and optical sensor are located out of body. Transdermal light delivery basically use long-wavelength visible light (>620 nm) or Near-Infrared (NIR) light (wavelength: ∼800 nm) because of optical attenuation in biological tissues (Nomoto et al., 2014). The restriction in wavelength range decreases the range of medical tools, which are developed to work with high optical system energies (Haruta et al., 2014). Therefore, transdermal light delivery approach is not suitable for neuron activity analyzing in deep regions of body. One of the advantages of this method is the stable setup and controllable location near the target and out of body.

Fiber optic approaches can achieve the minimum invasive delivery of light into the body tissues (O’Sullivan et al., 2009; Ohta, 2007; Ohta et al., 2006). As shown in Figure 2(b), this system needs optical source (a laser pulsed or white light), optical sensor (detector) to detect light, optical fibers to deliver light from light source to the optical source, and optical filters to filter the unwanted lights. The optical detector and light source are placed outside of the body. These approaches involve the use of high energy light such as blue light; though, optical fibers have not enough biocompatibility to biological tissues, because of imposing mechanical stress to the tissues during implantation caused by the inflexibility of fibers. This limitation makes it incompatible for long-term implantation (Ohta, Tokuda, Sasagawa, & Noda, 2009). In addition, light-guiding hydrogels (Ohta et al., 2006) and flexible-film waveguides (O’Sullivan et al., 2009) are applicable for long-term implantation. In fiber optic method optical source and optical sensor are located out of body; optical source sends light by optical fiber and optical sensor receives reflected light from tissues. Although, by using transdermal or fiber optic approach, it is difficult to monitor neural activity in deep regions of the brain, these approaches can perform the imaging of the brain surface.

In order to take the deep brain images, semiconductor devices have been developed, as shown in Figure 2(c), which include optical devices such as light sources (Light-Emitting Diodes [LEDs]) and detectors (image sensors). The mentioned devices are proper for long-term implantation with minimum invasiveness into the biological tissues due the miniaturization of LEDs and CMOS Image Sensors (CISs) to the small size (near 1 mm or smaller) (Haruta et al., 2014). Semiconductor devices have a number of significant advantages such as light weight, small dimensions of imaging system for free moving, integration of readout circuits and A/D converter, (Orlov, Szombathy, Chaudhry, & Haffajee, 2009) energy transfer system (Pang, Lee, & Suh, 2013), low power consumption, fully implantable light sources, and optical detectors. Hence, the mentioned devices are introduced as the potential devices to monitor neural activity in deep regions of the brain. However, semiconductor devices have some restrictions such as infection prevention, being floated or lost in other part of body, and difficulty to provide necassry power consumption of the device.

## 4. Image Sensor Description

**Figure 3:**
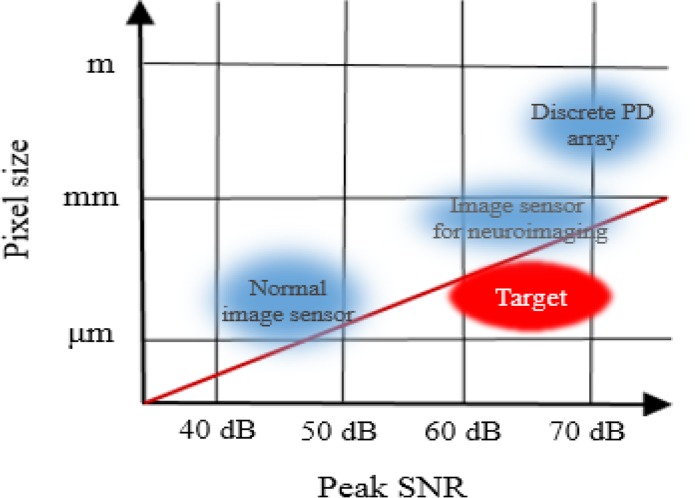
Target area of ideal SNR implantable image sensors (Sasagawa, et al., 2016)

As mentioned in section 3, the imaging system has an optical sensor (image sensor). The image sensor includes pixel arrays and readout circuits, and in most cases, the pixel circuitry is based on a 3-transistor type active pixel sensor (3T-APS) which is described in this section. Each pixel contains an n-well / p-substrate based photodiode (Dupret et al., 2011; Haruta et al., 2014; Hayami et al., 2016; Ho et al., 2014). The pixel size for some of the sensors, implanted in mouse’s brain, is designed to be 7.5 μm×7.5 μm or 15 μm×15 μm in different groups. The number of pixels is variable and depends on the necessary imaging area and required spatial resolution. The operating voltage of some image sensors which are implanted, are 3.3 V. Biosensor applications such as neural monitoring require a high Signal to Nose Ratio (SNR) of about 60–70 dB (Hayami et al., 2016) to detect small intensity change and intrinsic signal, as shown in Figure 3.

SNR is the ratio between the signal and noise and is used in imaging as a physical measure of the sensitivity of the imaging system. Implantable CMOS image sensor with self-resetting pixel structure is designed to decrease the photon shot noise and realize high SNR performance of brain imaging (Hayami et al., 2016). Moreover, implantable CMOS image sensor is fabricated and implanted into a rat brain (Hayami et al., 2016). Monitoring of brain activity can determine velocity of the blood-flow in the vessels of brain (Koppa et al., 2011).

**Figure 4:**
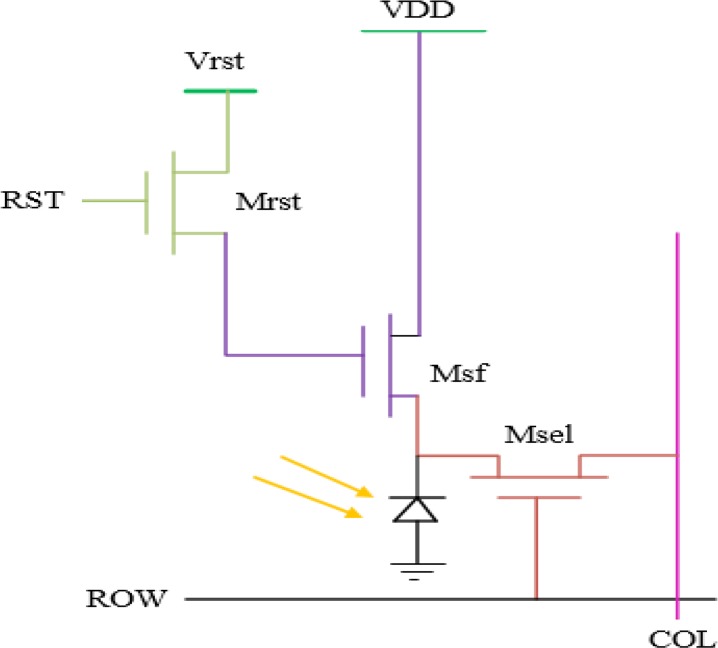
Schematic diagram of three-transistor Active Pixel Sensor

The used self-resetting pixel circuit is based on 3-transistor active pixel sensor and utilizes a Schmitt trigger inverter for self-resetting function. The Active Pixel Sensor (APS) structure consists of three transistors as shown in Figure 4. One transistor (Mrst) is used to reset floating diffusion (photodiode), the second one (Msf) is a source follower transistor and used as buffer and the last transistor is select transistor (Msel) that allows just one of the rows of the pixel array to be monitored simultaneously by the read-out circuits (Sasagawa et al., 2011). One of the advantages of the self-resetting sensors is the high SNR by the pixel saturation prevention. The pixel saturation is avoided by self-resetting mechanism, and can achieve a peak SNR of nearly 60 dB at a high light intensity. It should be noted that the maximum SNR of a conventional image sensor pixel, such as an Active Pixel Sensor (APS) is typically 40–50 dB (Braun & Fromherz, 2004; Sasagawa et al., 2010; Ahmadi & Jullien, 2009; Hayami et al., 2016). The results of self-resetting implantable CMOS image sensor show that the temporal and intrinsic signal changes in a living mouse brain tissues can be observed.

Another important feature of implantable image sensor is the power consumption, since high power consumption can generate high amount of heat (∼ 4W/cm^2^) which damages biological tissues (Haruta et al., 2014). It was reported that self-reset image sensor can achieve a peak SNR of nearly 60 dB at a very high light intensity, although the sensor consumed a relatively high power (nearly 185 mW) compared with (Ahmadi & Jullien, 2009). Furthermore, the same research group reported another CMOS self-reset image sensor developed to reduce the power consumption (nearly 20 mW) with a few changes in the inverter for self-resetting functionality with the similar concept of self-resetting function as in (Hayami et al., 2016). It should also be noted that the limitations of power consumption and heat generation has decreased in (Ahmadi & Jullien, 2009). To reduce the power consumption, intra-body communication technique is developed, which reduces power consumption and device size (Tagawa et al., 2009; Takehara et al., 2016). Intra-body communication is a wireless data transmission technique, where an imaging system transmits electric signals through body tissues (Tokuda et al., 2014; Tokuda, Takehara, Noda, Sasagawa, & Ohta, 2016; Yamagiwa, Ishida, & Kawano, 2015; Yamaguchi et al., 2016).

One of the challenging issues in CISs is the Dynamic Range (DR), which is defined as the ratio of largest non-saturating photocurrent to the smallest detectable signal (Ye, Daoud-El Baba, Peng, & Fussenegger, 2011). This feature determines the sensing performance range of the implantable image sensor to monitor small neural activity (Ahmadi & Jullien, 2009). Image sensors need high DR (nearly 88 dB) (Yuan, Chan, Fung, & Liu, 2009) for imaging and intrinsic signal detection biological tissues, which is challenging for a conventional image sensor. A pixel with the self-reset function realizes a high DR of light intensity to detect small changes in signals (Park et al., 2007; Ruiz & Shimamoto, 2006). For increasing DR, charge of full-well capacity of the pixel should be increased. Self-reset capability function increases the effective full-well capacity itself and can detect small changes which result in wide DR (Ye et al., 2011).

Another important aspect of image sensors is the fill factor of a pixel that describes the ratio of light sensitive area versus total area of the pixel implemented on the chip (Braun & Fromherz, 2004). Large readout circuit and more transistors result in reducing photosensitive area and low fill factor. In self-resetting image sensor, the sensor pixels include counter circuits and have a low fill factor. Although (Hayami et al., 2016) realized a self-resetting pixel without the reset counter, by simplifying the pixel circuit, the size of the pixel reduces and fill factor increases (nearly 31%). Moreover, (Ahmadi & Jullien, 2009) realized self-resetting pixel without a reset counter with fill factor of 26% by light emission energy reduction to reduce the system power consumption (Zrenner, 2002). CMOS implantable imaging device with fill factor range of 29%–35% with changing chip size, number of pixels, and pixel array size is also reported.

## 5. Comparison

Table 1 summarizes the specifications of recent implantable image sensors for biomedical application. As shown in Table 1, most of the implantable image sensors are fabricated in 0.35 μm CMOS technology due to the advantages of this technology such as low power consumption, small pixel sizes, and low leakage current. The small pixels sizes (nearly 7.5 μm×7.5 μm to 15 μm×15 μm) can improve spatial resolution, though in self-resetting implantable image sensor pixel size is 15 μm×15 μm due to Schmitt trigger circuit. As shown in Table 1, pixels facilitated with large readout circuit area result in low fill factor. Tree-transistor APS structure is used for most implantable image sensor, since it has lower noise and fewer transistors which can increase fill factor of the pixels. For practical purposes some main issues of implantable image sensor should be considered such as mechanical distortion of implanted device due to growth or death of living tissues, breaking implanted device due to stress from the biological tissues, causing stress to the living tissues from implanted device and distortion tissues, dissolving packaging materials into living tissues and damaged cells (Zhang & Murphy, 2007). Considering the mentioned issues and Table 1, in order to monitor neural activity in the deep brain, it is necessary for the implantable image sensor to have high SNR (above 60 dB) and high DR (about 88 dB) and low power consumption (less than safety threshold 4W/cm ^2^).

**Table 1.** Comparison of implantable image sensor specifications in different works

**Author**	**Kobayashi et al. (2014)**	**Haruta et al. (2014)**	**Sasagawa et al. (2010)**	**Sasagawa et al. (2016)**	**Yamaguchi et al. (2016)**
Technology (μm)	0.35	0.35	0.35	0.35	0.35
Pixel array	60×60	900×1920	176×144	60 H×134 V	60 H×20 V
Peak SNR (dB)	-	-	-	59	64
Pixel type	3-Tr APS	3-Tr APS	3-Tr APS	3-Tr APS	3-Tr APS
Dynamic range (dB)	-	-	66	High	Hhigh
Fill factor (%)	35	44	30	31	26
Chip size (μm×μm)	570×850 μm^2^	1048.6×2700 μm^2^	2×2.2 mm^2^	1H×2.7 Vmm^2^	1050H×3000 Vμm^2^
Pixel size (μm×μm)	7.5×7.5 μm^2^	7.5×7.5 μm^2^	7.5×7.5 μm^2^	15H×15 Vμm^2^	15H×15 Vμm^2^
Frame rate (Hz)	71.5	58	-	300	40.6
Power consumption (mW)	-	-	-	185	20
Weight (g)	-	0.02	-	0.02	0.02
Transistor per pixel	-	-	-	10	11
Photodiode	n-well/p-sub	n-well/p-sub	n-well/p-sub	n-well/p-sub	n-well/p-sub

In addition, a polyimide layer is normally coated on the entire device to protect it from body fluids and make it water-proof (Hayami et al., 2016). Furthermore, the image sensor and LEDs are placed on a flexible circuit substrate which makes it easier to get into the body (Zhang & Murphy, 2007). Using CMOS-based semiconductor fabrication technologies, the size of an image sensor can be relatively small (nearly 1 mm) (Ahmadi & Jullien, 2009). As the push for deeper submicron technologies in electronic industries advances, the pixel size for conventional CISs will further reduce (Sasagawa et al., 2011).

In the case of implantable neural system monitoring, the imaging device should be miniaturized with light weight to make experiments less invasive with freely moving animals. Capitalizing on this advantage, the imaging resolution is expected to improve for observing neural activity, though it is difficult to obtain high spatial resolution.

The spatial resolution is the ability of the image sensor to differentiate two objects which are close together (in space), and for increasing the spatial resolution, pixels’ size should decrease and pixels’ number increase that result in more power consumption and biological tissue damage due to temperature increase. Thus to address low spatial resolution developing implantable image sensor with light guide array plate should be performed to increase spatial resolution of imaging. Furthermore, the image sensor should have high SNR in order to achieve high resolution image and detect small changes in biological tissues (Ahmadi & Jullien, 2009; Hayami et al., 2009; Zrenner, 2002).

## 6. Conclusion

Implantable image sensors are beneficial for biomedical applications and there still exist a lot of issues to be optimized such as rising injection light intensity to increase image SNR without higher power consumption. In this paper, a number of key features were presented as the advantages of implantable image sensors toward the development of a highly sensitive sensor. Looking at the level of integration so far, the potential of such devices for other applications such as treatment of brain disorders and cancer diagnosis are demonstrated.

Different challenges for the implantable CIS are concluded, such as increasing the spatial resolution requires using more pixels resulting in more power consumption that damages biological tissues. The next generation of implantable image sensor should overcome the issues which mentioned in section 5 and the pixel size should shrink to increase the spatial resolution to achieve high DR (above 88 dB) and SNR (above 60 dB) to detect small intensity change and intrinsic signal. Although several issues should be resolved in terms of biocompatibility and durability for a long-term operation such as tissue infections, the loss of neurons and power delivery, increase tightly of implanted device to the biological tissues, packaging of chip with the most water-resistant and least damage to the body. Moreover, it is obvious that implantable CIS will play an important role for the next generation biomedical applications due to lightweight (as 0.02 g which is about 1/1000 of the weight of an adult mouse) and small size (near 1 mm or smaller) to implant in tissues and imaging various parts of the body.

## Ethical Considerations
